# Depression, anxiety, and post-traumatic stress disorder in association with cardiovascular disease among patients with systemic lupus erythematosus and rheumatoid arthritis in the All of Us Research Program

**DOI:** 10.1007/s10067-026-07942-1

**Published:** 2026-01-16

**Authors:** Jeong Yee, Emily G. Oakes, Leah Santacroce, May Y. Choi, Elizabeth W. Karlson, Karestan Koenen, Laura D. Kubzansky, Jing Cui, Candace H. Feldman, Karen H. Costenbader

**Affiliations:** 1 Division of Rheumatology, Department of Medicine, Division of Rheumatology, Inflammation and Immunity, Brigham and Women’s Hospital, Harvard Medical School, Boston, MA USA; 2 School of Pharmacy, Sungkyunkwan University, 2066 Seobu-Ro, Jangan-Gu, Suwon, 16419 Republic of Korea; 3 Cumming School of Medicine, University of Calgary, Calgary, AB Canada; 4 Department of Social and Behavioral Sciences, Department of Epidemiology, Harvard T.H. Chan School of Public Health, Boston, MA USA

**Keywords:** Cardiovascular disease, Depression, Mental health, Rheumatoid arthritis, Systemic lupus erythematosus

## Abstract

**Objectives:**

Both mental health conditions, such as depression, anxiety, and post-traumatic stress disorder (PTSD), and cardiovascular disease (CVD) events are increased in patients with rheumatoid arthritis (RA) and systemic lupus erythematosus (SLE). We assessed associations and interactions between mental health conditions, RA and/or SLE (RA/SLE), and CVD risk.

**Method:**

Patients with RA/SLE in All of Us Research Program were matched 1:20 to patients without either. We calculated hazard ratios (HR, 95% confidence intervals) for associations of baseline depression, anxiety, or PTSD with incident CVD, adjusting for socioeconomic and comorbid factors, repeating for RA and SLE separately. We tested for interactions between RA/ SLE and mental health conditions influencing CVD risk.

**Results:**

Among 5,543 patients with RA/SLE, matched to 110,860 patients without RA/SLE, 31.7% vs. 15.2% had mental health conditions. Matching factor-adjusted CVD event HR was the highest among those with RA/SLE and mental health conditions vs. those with neither (2.91, 2.52–3.36). After full adjustment, this decreased to 1.59 (1.37–1.85). While similar associations were observed for RA and SLE, higher risks were among patients with mental health conditions and SLE (2.08, 1.60–2.71) vs. RA (1.41, 1.17–1.70). No statistical interactions between mental health conditions and RA/SLE influencing CVD risks were detected.

**Conclusions:**

In this large US cohort, patients with RA/SLE and mental health conditions had the highest CVD risks compared to those with either condition or none. Strategies are needed to address mental health conditions that contribute to excess CVD risk for patients with RA/SLE.

**Table:** 

**Key points** • *Patients with RA/SLE and mental health conditions had the highest CVD incidence, compared to those with either RA/SLE or any of the mental health conditions alone.* • *Adjustment for sociodemographic factors and comorbidities attenuated this relative CVD risk.* • *No significant interactions were found between RA/SLE and mental health conditions influencing observed CVD risks.*

**Supplementary Information:**

The online version contains supplementary material available at 10.1007/s10067-026-07942-1.

## Introduction

Mental health conditions such as depression, anxiety, and post-traumatic stress disorder (PTSD) are increasingly recognized as cardiovascular disease (CVD) risk factors [1]. These conditions contribute to CVD through heightened stress responses, chronic inflammation, and unhealthy lifestyle behaviors such as smoking and obesity [2, 3].

Rheumatoid arthritis (RA) and systemic lupus erythematosus (SLE) are chronic systemic autoimmune diseases that predominantly affect females and are characterized by inflammation of the joints and multiple organ systems, along with a notably high CVD risk [4, 5]. The systemic inflammation associated with these diseases can induce endothelial dysfunction, thrombosis, and other pathophysiological changes that increase the risk of CVD [5, 6]. Depression, anxiety, and PTSD are also more prevalent among patients with RA and/or SLE (RA/SLE), which have often been attributed to living with a disabling disease and to systemic effects such as fatigue, myalgia, arthritis, and inflammation [7, 8].

Recent studies have suggested that inflammation-driven mental health conditions may share mechanistic pathways with CVD risk, particularly through inflammatory cytokines such as interleukin-6 (IL-6) and tumor necrosis factor (TNF)-α [9–11]. However, it remains unclear whether the coexistence of both RA/SLE and mental health conditions amplifies CVD risk. Therefore, considering whether mental health conditions interact with RA/SLE in influencing the risk of CVD, we aimed to assess their association with CVD risk among patients with RA/SLE, and to compare these associations with a large, matched population of patients without RA/SLE, within a large, diverse US cohort.

## Materials and methods

### Study population

The All of Us Research Program is a nationwide longitudinal cohort study aiming to enroll 1 million diverse participants across the USA. The detailed protocol for All of Us has been previously outlined [12, 13]. The operational protocol received approval from both its institutional review board (IRB) and our healthcare system's IRB, and all participants gave informed consent during enrollment. In brief, adults aged 18 and older were invited to participate through healthcare provider organizations affiliated with All of Us, directly via the All of Us website, or at specific events. After enrollment, participants completed several surveys, available in both English and Spanish at a fifth-grade reading level, each designed to take about 15 min. They also agreed to share their electronic health records (EHR), undergo physical measurements, and donate biospecimens.

Among the participants with baseline survey and linked EHR data in All of Us version 8 (released Feb 2025), patients with RA/SLE were identified by at least two International Classification of Diseases (ICD)−9, ICD-10, and Systematized Nomenclature of Medicine (SNOMED) codes on distinct dates during the baseline period [14, 15]. Baseline period was defined as 2 years before their enrollment. We excluded patients who had the CVD outcomes of interest (i.e., acute myocardial infarction (MI), stroke, heart failure, and procedures of percutaneous coronary intervention (PCI) or coronary artery bypass grafting (CABG)) before enrollment or who developed RA/SLE during the follow-up period. Each patient with RA/SLE was matched to 20 participants without any evidence of RA/SLE on age, biologic sex at birth, self-reported race/ethnicity, and enrollment year using nearest-neighbor matching algorithms. We selected 20 matched controls per case based on the availability of eligible controls and to achieve adequate statistical power while preserving balanced matching on these key variables.

### Baseline variables

From the baseline survey in All of Us, the following data were extracted: age, biologic sex, self-reported race and ethnicity, household income, education level, and smoking status. The Nationwide Community Deprivation Index is an area-level composite measure of neighborhood socioeconomic disadvantage based on poverty, income, education, insurance, public assistance, and vacant housing. It was derived from the US Census American Community Survey measures and calculated as the population-weighted average of the index for the US Census tracts based on their three-digit zip code [16]. Obesity was defined by body mass index (BMI) ≥ 30 kg/m^2^ from the baseline in-person physical measurement data. The Charlson Comorbidity Index (CCI) and CVD-related comorbidity were assessed using ICD-9, ICD-10, and SNOMED codes from the EHR during the baseline period [17]. Lupus nephritis was defined as SLE patients with nephritis [18]. Medication history was collected using RxNorm from the EHR during the baseline period. Patients were classified as taking a baseline medication if they had one or more prescriptions in this two-year period.

### Exposures and outcomes

A composite of three mental health conditions—depression, anxiety, and PTSD—was defined based on participants’ diagnoses; for each condition, a diagnosis was considered to be present if participants had at least two ICD-9/10 and SNOMED codes for that condition in the baseline period [19–21]. Thus, patients with one or more conditions were categorized as having any mental health conditions, allowing for overlapping diagnoses. The primary outcome was a composite of CVD events, including MI, stroke, heart failure (identified by ICD-9/10 and SNOMED codes), and CVD procedures of PCI/CABG (identified by CPT-4 codes) [22–25]. The codes used to define the primary outcome are summarized in Supplementary Table 1. Follow-up periods were defined as the time from enrollment until the first CVD event, through 5 years, or until the end of the study period (Oct 1, 2023).

### Statistical analyses

We analyzed the baseline variables among the patients with and without RA/SLE and with and without any of the three mental health conditions descriptively. To assess the associations of mental health conditions and of RA/SLE on CVD outcomes, we analyzed four groups: (1) participants without mental health conditions without RA/SLE (reference group), (2) those without mental health conditions and with RA/SLE, (3) those with mental health conditions without RA/SLE, and (4) those with mental health conditions and RA/SLE. We estimated incidence rates (IRs) and incidence rate ratios (IRRs) and plotted unadjusted cumulative incidence curves for CVD events over time across the four groups.

Adjusted Cox regression models compared the risks of CVD events among four groups (reference: those without RA/SLE or any mental health conditions at baseline). The base model adjusted for matching factors (i.e., age, sex, race/ethnicity, enrollment year). We conducted a first, socioeconomically adjusted model, additionally including annual household income, education level, and the Nationwide Community Deprivation Index. Then, our second model further adjusted for comorbidities, including smoking, obesity, the Charlson Comorbidity Index (calculated using 16 comorbidities, excluding rheumatic disease), hypertension, diabetes, dyslipidemia, and renal disease.

Subgroup analyses were performed by dividing the population into two groups: one with RA and matched controls, and the other with SLE and matched controls. Patients were classified as RA or SLE according to the majority of their billing codes, if they had both. A subgroup analysis, stratified by males and females, was also conducted. In sensitivity analyses, the secondary outcome was more narrowly defined as a composite of MI and stroke and was further expanded to include all-cause mortality (cause-specific mortality was not available).

To assess potential joint effects of having both mental health conditions and RA/SLE, we further tested for interactions between RA/SLE and mental health conditions with respect to CVD risk. Additive interactions were tested using the relative excess risk due to interaction (RERI), the proportion attributable to interaction (AP), and the synergy index (S). Interactions on the multiplicative scale were calculated using the cross-product of the two risk factors and the “interactionR” package. A *p*-value < 0.05 was considered statistically significant for all analyses, and 95% confidence intervals were also examined for significance. All statistical analyses were performed using R version 4.1.0 in a Jupyter Notebook contained in the All of Us workbench.

## Results

### Baseline characteristics

We studied 5543 patients with RA/SLE and 110,860 matched participants without RA/SLE. Among them, 1758 (31.7%) and 16,823 (15.2%) had depression, anxiety, and/or PTSD (Table 1). Among both those with and without RA/SLE, patients with ≥ 1 mental health condition were more likely to be less educated, to be in a low-income group, to have ever smoked, to be obese, and to have multiple comorbidities and to receive more treatment, than those with none of these mental health conditions. Sixty-seven percent of the RA/SLE patients with mental health conditions vs. 56.5% of those without mental health conditions were taking glucocorticoids at baseline.

**Table 1:** Baseline demographic and survey data from patients according to mental health condition diagnoses and diagnoses of rheumatoid arthritis (RA) or systemic lupus erythematosus (SLE) in the *All of Us* Research Program (data release v8)

Characteristics	Patients with RA or SLE^a^		Patients without RA or SLE	
	Patients with mental health conditions (*n* = 1758; 31.7%)	Patients without mental health conditions (*n* = 3785; 68.0%)	Patients with mental health conditions (*n* = 16,823; 15.2%)	Patients without mental health conditions (*n* = 94,037; 84.8%)
Age, years	54.4 ± 13.4	56.5 ± 14.8	55.2 ± 13.6	55.9 ± 14.6
Self-reported sex (%)				
Female	1536 (87.4)	3116 (82.3)	14,793 (87.9)	78,341 (83.3)
Male	222 (12.6)	669 (17.7)	2030 (12.1)	15,696 (16.7)
Self-reported race and ethnicity (%)				
Hispanic Americans	317 (18.0)	772 (20.4)	2978 (17.7)	18,696 (19.9)
Non-Hispanic Black Americans	280 (15.9)	659 (17.4)	2457 (14.6)	16,077 (17.1)
Non-Hispanic White Americans	958 (54.5)	1937 (51.2)	9668 (57.5)	48,882 (52.0)
Non-Hispanic others/prefer not to answer/missing	203 (11.5)	417 (11.0)	1720 (10.2)	10,382 (11.0)
Enrollment year				
2018	447 (25.4)	854 (22.6)	4133 (24.6)	22,026 (23.4)
2019	498 (28.3)	1152 (30.4)	5055 (30.0)	28,832 (30.7)
2020	136 (7.7)	357 (9.4)	1479 (8.8)	8183 (8.7)
2021	185 (10.5)	475 (12.5)	1921 (11.4)	10,681 (11.4)
2022	270 (15.4)	514 (13.6)	2273 (13.5)	13,165 (14.0)
2023	222 (12.6)	433 (11.4)	1962 (11.7)	11,150 (11.9)
Annual household income, $				
< 35 k	780 (44.4)	1146 (30.3)	6273 (37.3)	26,822 (28.5)
35 k– < 100 k	432 (24.6)	1037 (27.4)	4663 (27.7)	26,288 (28.0)
≥ 100 k	203 (11.5)	808 (21.3)	2720 (16.2)	22,037 (23.4)
Prefer not to answer/missing	343 (19.5)	794 (21.0)	3167 (18.8)	18,890 (20.1)
Education level				
High school graduate or less	502 (28.6)	954 (25.2)	4859 (28.9)	24,187 (25.7)
Some college	629 (35.8)	1119 (29.6)	5061 (30.1)	24,170 (25.7)
College graduate or above	591 (33.6)	1649 (43.6)	6541 (38.9)	43,741 (46.5)
Prefer not to answer/missing	36 (2.0)	63 (1.7)	362 (2.2)	1939 (2.1)
Nationwide Community Deprivation Index^b^	0.3 ± 0.1	0.3 ± 0.1	0.3 ± 0.1	0.3 ± 0.1
Smoking				
Ever	796 (45.3)	1418 (37.5)	7513 (44.7)	34,458 (36.6)
Never	914 (52.0)	2269 (59.9)	8843 (52.6)	56,603 (60.2)
Missing	48 (2.7)	98 (2.6)	467 (2.8)	2976 (3.2)
BMI (kg/m^2^)				
≥ 30	896 (51.0)	1564 (41.3)	8376 (49.8)	38,122 (40.5)
< 30	732 (41.6)	1998 (52.8)	7562 (45.0)	49,669 (52.8)
Missing	130 (7.4)	223 (5.9)	885 (5.3)	6246 (6.6)
Mental health conditions				
Depression	1353 (77.0)	-	12,143 (72.2)	-
Anxiety	1071 (60.9)	-	9962 (59.2)	-
PTSD	196 (11.1)	-	1856 (11.0)	-
Charlson comorbidity index^c^				
0	644 (36.6)	2098 (55.4)	7775 (46.2)	73,868 (78.6)
1	481 (27.4)	741 (19.6)	3722 (22.1)	9047 (9.6)
2 +	633 (36.0)	946 (25.0)	5326 (31.7)	11,122 (11.8)
Comorbidities				
Hypertension	900 (51.2)	1400 (37.0)	7639 (45.4)	17,289 (18.4)
Diabetes	408 (23.2)	566 (15.0)	3662 (21.8)	8086 (8.6)
Dyslipidemia	650 (37.0)	963 (25.4)	6275 (37.3)	14,110 (15.0)
Renal disease	192 (10.9)	309 (8.2)	936 (5.6)	2152 (2.3)
Lupus nephritis^d^	141 (8.0)	277 (7.3)	-	-
Medications				
Conventional DMARDs	841 (47.8)	1921 (50.8)	305 (1.8)	988 (1.1)
Biologic and targeted synthetic DMARDs	398 (22.6)	868 (22.9)	223 (1.3)	698 (0.7)
Other immunosuppressants	250 (14.2)	480 (12.7)	539 (3.2)	1696 (1.8)
Glucocorticoids	1178 (67.0)	2138 (56.5)	6809 (40.5)	19,425 (20.7)
Antidepressants	1207 (68.7)	844 (22.3)	11,145 (66.2)	11,863 (12.6)
Anxiolytics	757 (43.1)	563 (14.9)	6075 (36.1)	7931 (8.4)
Antipsychotics	466 (26.5)	354 (9.4)	3638 (21.6)	5393 (5.7)
ACEI/ARBs	450 (25.6)	824 (21.8)	4111 (24.4)	12,704 (13.5)
Beta-blockers	505 (28.7)	793 (21.0)	3841 (22.8)	10,876 (11.6)
Calcium channel blockers	381 (21.7)	636 (16.8)	2768 (16.5)	8109 (8.6)
Diuretics	440 (25.0)	722 (19.1)	3644 (21.7)	10,621 (11.3)
Aspirin	393 (22.4)	583 (15.4)	2846 (16.9)	7737 (8.2)
Statins	426 (24.2)	854 (22.6)	4878 (29.0)	14,273 (15.2)

*ACEI*, angiotensin-converting enzyme inhibitors; *ARB*, angiotensin II receptor blocker; *BMI*, body mass index; *CVD*, cardiovascular disease; *DMARD*, disease modifying anti-rheumatic drug; *PTSD*, post-traumatic stress disorder. According to All of Us Research Program data sharing policies, cells with fewer than 20 participants were suppressed

^a^There were 3772 patients with RA only, 1533 with SLE only, and 238 patients with both RA and SLE

^b^Nationwide Community Deprivation Index was derived from the US Census American Community Survey based on three-digit zip code. There were 15,572 participants with missing data

^c^Charlson comorbidity index calculated using 16 comorbidities, excluding rheumatic disease

^d^Lupus nephritis was defined as SLE patients with nephritis. (Chibnik L et al., *Lupus*, 2010)

### Associations of RA/SLE and mental health conditions with CVD risk

**Fig. 1:**
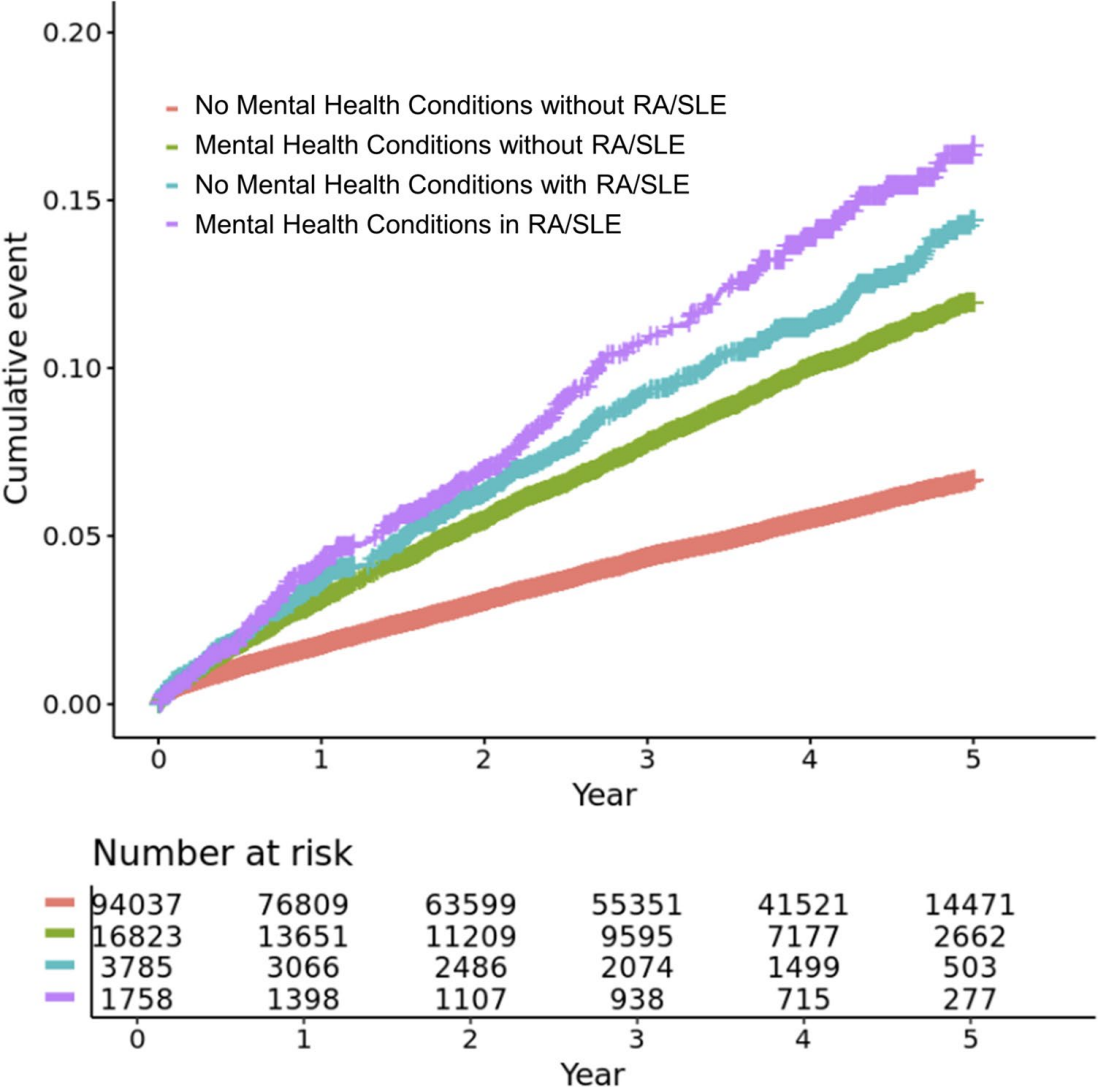
Unadjusted cumulative incidence curves for time to major cardiovascular events for patients according to mental health condition diagnoses and rheumatoid arthritis (RA) or systemic lupus erythematosus (SLE) diagnoses in the All of Us Research Program (data release version 8)

Patients were followed from baseline for a mean of 3.11 years (SD 1.69). We found 4253 events that occurred over 293,512 person-years among patients with neither RA/SLE nor any mental health condition (IR 14.5/1000 person-years, reference group; Table 2); 1372 events over 51,540 person-years among patients with a mental health condition but without RA/SLE (IR 26.6/1000 person-years; IRR 1.83 [95%CI 1.73–1.95]); and 357 events occurred over 11,332 person-years among patients with RA/SLE but without any mental health conditions (IR 31.5/1000 person-years; IRR 2.17 [95%CI 1.95–2.42]). Patients with RA/SLE and a mental health condition had the highest incidence of CVD events, with 194 events occurring over 5,196 person-years (IR 37.3/1000 person-years; IRR 2.58 [95%CI 2.23–2.98]). The unadjusted risks of CVD events in these matched populations were significantly higher among patients with both of these mental health conditions and among those with RA/SLE compared to those without, and highest among those with both sets of conditions (Fig. 1).

**Table 2:** Incidence rates of major cardiovascular events for patients according to mental health condition diagnoses and rheumatoid arthritis (RA) or systemic lupus erythematosus (SLE) diagnoses in the All of Us Research Program (data release version 8)

	No. of events	No. of person-years	Incidence rate per 1000 person-years (95% CI)	Incidence rate ratio (95%CI)
No mental health conditions among patients without RA/SLE	4253	293,512	14.5 (14.1–14.9)	1 (ref)
Mental health conditions among patients without RA/SLE	1372	51,540	26.6 (25.2–28.1)	1.83 (1.73–1.95)
No mental health conditions among patients with RA/SLE	357	11,332	31.5 (28.3–34.9)	2.17 (1.95–2.42)
Mental health conditions among patients with RA/SLE	194	5196	37.3 (32.3–43.0)	2.58 (2.23–2.98)

Compared to those with neither RA/SLE nor any mental health condition, the HRs for CVD events in the matched populations (additionally adjusting for matching factors to more finely account for population differences) were as follows: 2.00 (95% CI 1.88–2.12) for those with mental health conditions but without RA/SLE, 2.12 (95% CI 1.90–2.36) for those with RA/SLE but without mental health conditions, and 2.91 (95% CI 2.52–3.36) for patients with both mental health conditions and RA/SLE (Table 3). After adjusting for socioeconomic factors, these HRs were slightly reduced to 1.90 (95% CI 1.78–2.02), 2.06 (95% CI 1.85–2.31), and 2.68 (95% CI 2.31–3.12), but the difference between those with and without mental health conditions in RA/SLE remained statistically significant. After further adjustment for multiple comorbidities related to risk of CVD, the HRs for CVD events were decreased as follows: 1.24 (95% CI 1.16–1.32) for those with mental health conditions but without RA/SLE, 1.54 (95% CI 1.38–1.73) for those with RA/SLE but without mental health conditions, and 1.59 (95% CI 1.37–1.85) for those with both RA/SLE and mental health conditions.

**Table 3:** Hazard ratios for major cardiovascular events for patients according to mental health condition diagnoses and rheumatoid arthritis (RA) or systemic lupus erythematosus (SLE) diagnoses in the All of Us Research Program (data release version 8)

Patient population	Matched model HR (95%CI)	Model I: Socioeconomically adjusted HR (95%CI)	Model II: Additionally comorbidity adjusted HR (95%CI)
No mental health conditions among patients without RA/SLE	1 (ref)	1 (ref)	1 (ref)
Mental health conditions among patients without RA/SLE	2.00 (1.88–2.12)	1.90 (1.78–2.02)	1.24 (1.16–1.32)
No mental health conditions among patients with RA/SLE	2.12 (1.90–2.36)	2.06 (1.85–2.31)	1.54 (1.38–1.73)
Mental health conditions among patients with RA/SLE	2.91 (2.52–3.36)	2.68 (2.31–3.12)	1.59 (1.37–1.85)

Matched model, matched on and additionally adjusted for matching factors (age, sex, race/ethnicity, year of enrollment). Model I additionally adjusted for annual house income, education level, and Nationwide Community Deprivation Index. Model II additionally adjusted for smoking, obesity, Charlson Comorbidity Index (calculated with 16 comorbidities, excluding rheumatic disease), hypertension, diabetes, dyslipidemia, and renal diseases

### Subgroup analysis

In the subgroup analysis of patients with RA and their matched controls (Table 4), all HRs were significantly higher than the referent group of those with neither RA nor a mental health condition: the HR for individuals with mental health conditions but without RA was 1.28 (95% CI 1.18–1.38), for those with RA but without mental health conditions was 1.29 (95% CI 1.12–1.49), and for individuals with both RA and mental health conditions was 1.41 (95% CI 1.17–1.70). In our subgroup analysis focused on patients with SLE and their matched controls, all HRs were again increased compared to those without SLE or a mental health condition, but SLE patients’ CVD risks were higher. The HRs for those with SLE but without mental health conditions and for those with both SLE and mental health conditions were notably elevated: 2.20 (95% CI 1.81–2.68) and 2.08 (95% CI 1.60–2.71), respectively.

**Table 4:** Additional analyses and adjusted hazard ratios for major cardiovascular events for patients according to mental health condition diagnoses and rheumatoid arthritis (RA) or systemic lupus erythematosus (SLE) diagnoses in the All of Us Research Program (data release version 8)

Analysis	No mental health conditions among patients without RA/SLE	Mental health conditions among patients without RA/SLE	No mental health conditions among patients with RA/SLE	Mental health conditions among patients with RA/SLE
Subgroup analysis by diseases^a^				
RA (*n* = 3861) and matched controls (*n* = 77,220)	1 (ref)	1.28 (1.18–1.38)	1.29 (1.12–1.49)	1.41 (1.17–1.70)
SLE (*n* = 1657) and matched controls (*n* = 33,140)	1 (ref)	1.13 (0.98–1.30)	2.20 (1.81–2.68)	2.08 (1.60–2.71)
Subgroup analysis by sex				
Male (*n* = 18,617)	1 (ref)	1.27 (1.08–1.48)	1.29 (1.01–1.66)	1.21 (0.79–1.84)
Female (*n* = 97,786)	1 (ref)	1.23 (1.14–1.33)	1.63 (1.43–1.85)	1.68 (1.43–1.98)
Sensitivity analysis				
Outcome defined as a composite of MI and stroke	1 (ref)	1.33 (1.21–1.45)	1.63 (1.40–1.90)	1.92 (1.58–2.34)
Outcome defined as a composite of MI, stroke, HF, PCI/CABG, and all-cause mortality	1 (ref)	1.23 (1.15–1.30)	1.50 (1.34–1.66)	1.61 (1.39–1.85)

*HF*, heart failure; *MI*, myocardial infarction; *RA*, rheumatoid arthritis; *SLE*, systemic lupus erythematosus

Adjusted for matching factors (age, sex, race/ethnicity, year of enrollment), annual house income, education level, Nationwide Community Deprivation Index, smoking, obesity, Charlson Comorbidity Index (calculated with 16 comorbidities, excluding rheumatic disease), hypertension, diabetes, dyslipidemia, and renal diseases

^a^There were 3772 patients with RA only, 1533 with SLE only, and 238 patients with both RA and SLE. Among patients with both RA and SLE, they were assigned to the diagnosis with the higher number of codes. Those with an equal number of codes for both diseases (*n* = 25) were not included in the subgroup analysis

In sex-stratified analyses, we found the main associations, as predicted, were more pronounced among females (Table 4). Among males, the HR for patients with mental health conditions but without RA/SLE was 1.27 (95% CI 1.08, 1.48); for those with RA/SLE but without mental health conditions, 1.29 (95% CI 1.01, 1.66); and for those with both RA/SLE and mental health conditions, 1.21 (95% CI 0.79, 1.84). Among females, the corresponding HRs were higher: 1.23 (95% CI 1.14, 1.33), 1.63 (95% CI 1.43, 1.85), and 1.68 (95% CI 1.43, 1.98), respectively.

### Sensitivity analyses

The sensitivity analysis, restricted to a composite of stroke and MI, yielded results consistent with the main analysis (Table 4). The hazard ratios (HRs) were 1.33 (95% CI 1.21–1.45) for individuals with mental health conditions but without RA/SLE, 1.63 (95% CI 1.40–1.90) for those with RA/SLE but without mental health conditions, and 1.92 (95% CI 1.58–2.34) for individuals with both RA/SLE and mental health conditions. Furthermore, the associations remained robust in additional sensitivity analyses, which expanded the outcome to include all-cause mortality (Table 4).

### Interaction analysis

When analyzing the interaction between RA/SLE and mental health status on CVD risk, the RERI was non-significant at − 0.19 (95% CI − 0.49–0.11; *p* 0.89), AP was − 0.12 (95% CI − 0.32–0.08; *p* 0.12), and the synergy index was 0.76 (95% CI 0.48–1.20; *p* 0.88), all indicating no significant additive or synergistic interaction. Similarly, the multiplicative interaction was 0.83 (95% CI 0.69–1.01; *p* 0.06), demonstrating no significant interaction between having both RA/SLE and mental health conditions on CVD risk.

## Discussion

Over the past few decades, several studies have demonstrated the highly prevalent comorbidity of mental health conditions among patients with RA/SLE, demonstrated again in this large American cohort. Among patients with RA, mental health conditions have been found to be more common than in the general population, with prevalence estimates ranging from 14 to 48% [26]. Similarly, many studies and meta-analyses have shown the prevalence of major depression and anxiety to be elevated at 24% and 37% among cohorts of patients with SLE, substantially higher than those observed in the general population [8]. Our study revealed a similarly high prevalence of depression, anxiety, and PTSD, with 31.7% of patients with RA/SLE having at least one of these mental health conditions, nearly double the prevalence found in the general population matched on age, sex, self-reported race and ethnicity, and enrollment year matched general population.

Patients with RA/SLE, as well as those with mental health conditions, are at an increased risk of developing CVD. Previous studies have shown that the risk of CVD in RA/SLE patients is at least twice that in the general population, with relative risks especially high among young women who have low CVD risks in the absence of autoimmune rheumatic disease or any other strong risk factor [5, 27, 28]. Many past studies have demonstrated that patients with mental health conditions are at an increased risk for incident CVD, with relative risk estimates ranging from 1.5 to 1.8 [29]. The current large cohort study revealed that patients with both RA/SLE and mental health conditions had the highest risks of CVD, with an HR of almost 3 in matched analyses.

In analyses stratified by autoimmune rheumatic disease type, we found that the relative risks were higher among patients with SLE than among those with RA, consistent with prior literature showing the particularly high CVD burden in SLE [28, 30]. Nevertheless, the relative impact of depression and anxiety on CVD risk appeared similar in both diseases. Thus, although the relative CVD risk may differ between RA and SLE, the contribution of mental health conditions to this excess CVD risk was consistent in both RA and SLE.

In sex-stratified analyses, the observed associations were generally more pronounced among females than among males. RA/SLE, as well as mental health conditions such as depression, anxiety, and PTSD, are all much more common among females than among males, and young women with RA/SLE have dramatically increased relative risks of CVD events compared to age-matched individuals without these diseases [31–33]. These findings highlight the importance of considering sex differences when assessing CVD risk among patients with RA/SLE, particularly in the context of coexisting mental health conditions.

The links between RA/SLE, mental health conditions, and CVD risk have been studied, and the biological mechanisms appear to be complex and numerous, although increased systemic inflammation has emerged as a key shared mechanism [2, 27]. Past work has suggested that inflammatory markers such as IL-6 and C-reactive protein (CRP) are elevated in depression and mental health conditions [34]. These stress-related inflammatory changes contribute to endothelial dysfunction, dysregulation of autonomic and vascular functions, and accelerated CVD development [35]. Other immunologic alterations, particularly elevated proinflammatory cytokines like IL-1β, TNFα, and IL-6, are implicated in both conditions and may contribute to neuropsychiatric symptoms [36]. Chronic stress, hypertension, and elevated cortisol levels in mental health conditions also contribute to sustained inflammation, leading to endovascular damage and increased risk of myocardial infarction, stroke, and heart failure [37–39]. Furthermore, coexisting mental health conditions are associated with increased risk of CVD in patients with RA/SLE, potentially reflecting their relationship with medication-related cardiometabolic effects. This may involve more frequent or prolonged use of glucocorticoids, as shown in Table 1, along with NSAIDs, which are known to induce hypertension, insulin resistance, dyslipidemia, and increased thrombotic risk.

Our sequentially-adjusted models revealed that the elevated HR for CVD associated with mental health conditions in those with vs. without RA/SLE was somewhat attenuated after accounting for socioeconomic factors. HRs in all groups further declined after adjusting for multiple comorbidities (including smoking, obesity, hypertension, diabetes, hyperlipidemia, and renal disease, as well as the CCI), relative to the referent group without mental health conditions or RA/SLE. Thus, as in past studies, this suggests that a portion of the observed increased risk may be attributed to an excess of traditional cardiovascular risk factors that were more prevalent among those with RA/SLE and mental health conditions [39–41]. Patients with lower socioeconomic status, including lower annual income and education levels, often have limited access to healthcare, poorer living conditions, and higher stress levels, all of which negatively impact cardiovascular health [42–44].

No statistically significant synergistic interaction between RA/SLE and mental health conditions on CVD risk was observed. This could be explained by a ceiling effect, where the CVD risk is already substantially elevated in patients with RA/SLE, leaving limited additional risk that mental health conditions could confer when they are present. In addition, overlapping pathophysiological pathways, including systemic inflammation [2, 27], may contribute to CVD risk in patients with RA/SLE and mental health conditions, limiting our ability to detect synergistic effects.

We recognize the limitations of this study. Since patients were identified using diagnosis codes, misclassification errors are possible despite these diagnostic algorithms being well-established, validated, and commonly used in EHR datasets. In addition, disease activity of RA/SLE was not captured in this study, which may have limited a more detailed examination of how disease activity affects CVD outcomes in this population. Incorporating disease activity could provide further insights into the pathways connecting mental health and CVD outcomes. Similarly, drug prescriptions might be underreported due to potential gaps in the EHR records, and we captured only medication use, regardless of dose or treatment duration, as these data were incomplete. Furthermore, residual confounding due to unmeasured factors remains possible and could affect the interpretation of our findings. We observed baseline differences in medication use among the four populations, but we were not able to conduct advanced pharmacoepidemiologic analyses to investigate how these may mediate the observed differences in CVD risks. Additionally, physical exercise, dietary patterns, and medication adherence were not available, and may also have contributed to the confluence of risks. Lastly, we did not exclude patients with other autoimmune diseases; therefore, the presence of additional autoimmune conditions may have influenced the observed associations.

There are many strengths of this unique analysis. We examined the joint effects of two sets of known risk factors for incident CVD: mental health conditions and RA/SLE. We did this in a large US cohort with both self-reported data and longitudinal EHR record data and outcomes. We have found that both sets of risk factors elevate CVD risk to approximately the same extent, a doubling of risk, but the presence of both does not interact synergistically to further augment risk. After adjustment for sets of known traditional CVD risk factors, including sociodemographic factors and comorbidities, the risk associated with both mental health conditions and RA/SLE was attenuated, suggesting causal pathways that may be shared between these risk factors. Further work is needed to better identify modifiable risk factors for depression, anxiety, and PTSD, which are alarmingly prevalent and contribute to high risks of CVD among those with RA/SLE. Early identification through regular screening, along with management of mental health conditions using a multidisciplinary care approach involving rheumatology, psychiatry, and cardiology, is needed to mitigate CVD risk in patients with RA/SLE.

## Supplementary Information

Below is the link to the electronic supplementary material.

ESM1 (DOCX.23.6 KB)
