# Supplementary material for: Depression, anxiety, and post-traumatic stress disorder in association with cardiovascular disease among patients with systemic lupus erythematosus and rheumatoid arthritis in the All of Us Research Program
Source: Clin Rheumatol. 2026 Jan 16;45(2):791–801. doi: 10.1007/s10067-026-07942-1 (PMC12833825; doi:10.1007/s10067-026-07942-1)
Supplement: Supplementary file 1 — (DOCX.23.6 KB) [file 10067_2026_7942_MOESM1_ESM.docx]

| **Supplementary Table 1. Diagnoses and procedure codes for cardiovascular events** | | | | |
| --- | --- | --- | --- | --- |
| **Components** | **ICD-9** | **ICD-10** | **SNOMED** | **CPT-4** |
| Myocardial infarction | 410 | I21, I22 | 57054005 |  |
| Stroke | 362.3, 433.x1, 434.x1, 436, 430, 431 | H34.1, I63, I64, I60, I61 | 432504007, 1386000 |  |
| Heart failure | 428 | I50 | 84114007 |  |
| PCI |  |  |  | 92980-92984, 92995-92996, 92920-92944 |
| CABG |  |  |  | 33510-33519, 33521-33523, 33533-33536 |

| **Table 4. Additional analyses and adjusted hazard ratios for major cardiovascular events for patients according to mental health condition diagnoses and rheumatoid arthritis (RA) or systemic lupus erythematosus (SLE) diagnoses in the All of Us Research Program (data release version 8)** | | | | |
| --- | --- | --- | --- | --- |
| **Analysis** | **No mental health conditions among patients without RA/SLE** | **Mental health conditions among patients without RA/SLE** | **No mental health conditions among patients with RA/SLE** | **Mental health conditions among patients with RA/SLE** |
| **Subgroup analysis by diseases^a^** | | | | |
| **RA (n=3861) and matched controls (n=77220)** | 1 (ref) | 1.28 (1.18-1.38) | 1.29 (1.12-1.49) | 1.41 (1.17-1.70) |
| **SLE (n=1657) and matched controls (n=33140)** | 1 (ref) | 1.13 (0.98-1.30) | 2.20 (1.81-2.68) | 2.08 (1.60-2.71) |
| **Subgroup analysis by sex** | | | | |
| **Male (n=18617)** | 1 (ref) | 1.27 (1.08-1.48) | 1.29 (1.01-1.66) | 1.21 (0.79-1.84) |
| **Female (n= 97786)** | 1 (ref) | 1.23 (1.14-1.33) | 1.63 (1.43-1.85) | 1.68 (1.43-1.98) |
| **Sensitivity analysis** |  |  |  |  |
| **Outcome defined as a composite of MI and stroke** | 1 (ref) | 1.33 (1.21-1.45) | 1.63 (1.40-1.90) | 1.92 (1.58-2.34) |
| **Outcome defined as a composite of MI, stroke, HF, PCI/CABG, and all-cause mortality** | 1 (ref) | 1.23 (1.15-1.30) | 1.50 (1.34-1.66) | 1.61 (1.39-1.85) |
| HF: heart failure; MI: myocardial infarction; RA: rheumatoid arthritis; SLE: systemic lupus erythematosus.  Adjusted for matching factors (age, sex, race/ethnicity, year of enrollment), annual house income, education level, Nationwide Community Deprivation Index, smoking, obesity, Charlson Comorbidity Index (calculated with 16 comorbidities, excluding rheumatic disease), hypertension, diabetes, dyslipidemia, and renal diseases.  ^a^There were 3,772 patients with RA only, 1,533 with SLE only, and 238 patients with both RA and SLE. Among patients with both RA and SLE, **they** were assigned to the diagnosis with the higher number of codes. Those with an equal number of codes for both diseases (n=25) were not inlucded in the subgroup analysis. | | | | |
